# Changes in the diagnosis, treatment, and prognosis of multiple myeloma in southern Jiangxi Province from 2012 to 2022

**DOI:** 10.1097/MD.0000000000047332

**Published:** 2026-01-23

**Authors:** Qi Xiao, Pingfang Liu, Lunwang Liang, Jing Li, Xiqing Rao, Sizhe Zhu, Zoufang Huang

**Affiliations:** a Department of Hematology, The Endemic Disease (Thalassemia) Clinical Research Center of Jiangxi Province, First Affiliated Hospital of Gannan Medical University, Ganzhou, Jiangxi, China; b School of Medical Technology, Gannan Medical University, Ganzhou, Jiangxi, China; c School of the First Clinical Medicine, Gannan Medical University, Ganzhou, Jiangxi, China.

**Keywords:** clinical research, multiple myeloma, prognostic factors, treatment

## Abstract

A retrospective study was conducted to analyze changes in the diagnosis, treatment, and prognosis of multiple myeloma (MM) patients in a provincial medical center in the Gannan region and to describe the gradual improvements in these methods. The clinical data of patients with newly diagnosed multiple myeloma (NDMM) at the First Affiliated Hospital of Gannan Medical University from January 1, 2013, to December 31, 2022, were retrospectively collected. Demographic and clinical characteristics, 1st-line treatment and its efficiency, survival, and prognostic factors of patients with MM in the past 10 years were analyzed. A total of 439 patients with NDMM were identified, with a median age of 64 (33–91) years and a male-to-female ratio of approximately 1.26:1. Among the patients, 56.95% were classified as International Staging System stage III. A total of 168 patients (38.27%) underwent fluorescence in situ hybridization, with a positive rate of 68.45%. The proportion of patients who underwent autologous stem cell transplantation was low. The main regimen used for induction therapy was proteasome inhibitor + immunomodulatory drug-based triple chemotherapy. During follow-up, the overall response rate was 72.73%, and the complete response rate was 20.35%. The short-term and long-term overall survival (OS) rates increased annually. The median OS was 1273 days, and the 5-year OS% was 35.70%. Multivariate Cox analysis revealed that age > 65 years; no autologous stem cell transplantation; progression to relapsed and refractory MM; and increased lactate dehydrogenase, β_2_-microglobulin, and uric acid levels were independent poor prognostic factors affecting OS. In the past decade, the diagnosis of NDMM and treatment methods in the Gannan area have gradually improved. With the popularization of new technologies and the widespread use of standardized treatment regimens containing new drugs, the short-term and long-term survival of MM patients in the Gannan area has significantly improved.

## 1. Introduction

Multiple myeloma (MM) is a hematological malignancy caused by monoclonal proliferation of malignant plasma cells^[1,2]^ that still cannot be completely cured.^[3]^ With the development and application of new drugs such as proteasome inhibitors (PIs) and immunomodulatory drugs (IMiDs), monoclonal antibodies, cellular immunotherapy, and autologous stem cell transplantation (ASCT), as well as evaluation methods such as free light chain measurement, minimal residual disease detection, and biological examinations (chromosome testing and fluorescence in situ hybridization^[4]^), patient survival has significantly improved, with overall survival (OS) increasing from 3 years to 8 to 10 years.^[5,6]^ The uneven distribution of economic levels and medical resources worldwide has led to significant differences in treatment outcomes across different regions of the world.^[7]^ Compared with developed countries, the economic, technological, and infrastructure constraints of low-income and middle-income countries lead to a large gap in the prognosis of MM.^[8]^ Among the different races, the prognosis of Asian patients is relatively good; however, the prognosis of Chinese patients is poor, which is likely related to the late diagnosis of MM patients, late application of new drugs, and low rate of ASCT.^[9–11]^ The economic level of the southern region of Jiangxi Province has significantly improved since the implementation of the revitalization policies in the Soviet region and the rise of central China since 2012. This study analyzes the clinical characteristics and outcomes of 439 newly diagnosed multiple myeloma (NDMM) patients from a single center in Gannan over the past decade. It highlights disparities in diagnosis, treatment, and survival compared to major Chinese cities. The findings aim to inform future management strategies for NDMM in this region.

## 2. Materials and methods

### 2.1. Patients

In this study, 439 patients with NDMM who were diagnosed and treated at the First Affiliated Hospital of Gannan Medical University from January 2012 to December 2022 were retrospectively enrolled. MM patients were diagnosed according to the International Myeloma Working Group diagnostic criteria.^[12]^ All the data were collected and analyzed after informed consent forms were signed by the patients or their agents.

### 2.2. Clinical data

Data, including demographics, clinical characteristics (including white blood cell and platelet^[13]^ counts; hemoglobin, serum calcium,^[14]^ creatinine, urea, serum albumin, β_2_-microglobulin [β_2_-MG], and lactate dehydrogenase [LDH] levels; serum immunofixation electrophoresis results; bone marrow cell morphology; bone marrow biopsy results; flow cytometry results; chromosome examination results; cytogenetic changes detected by FISH; imaging examination results; extramedullary lesion evaluation; and mild amyloidosis), 1st-line treatment regimens, maintenance treatment plans and durations, response rates, and survival rates, were collected from patients’ electronic medical records. Extramedullary disease was diagnosed with positron emission tomography/computed tomography (CT) scan, CT scan, or magnetic resonance imaging.

### 2.3. Evaluation standards

Patient responses were characterized according to the International Myeloma Working Group consensus criteria for response from 2016 as follows^[12]^: strict complete remission (sCR), complete remission (CR), very good partial remission (VGPR), partial remission (PR), minimal remission, stable disease, and disease progression. The overall response rate was calculated as the sum of the CR rates (sCR + CR), VGPR rate, and PR rate.

### 2.4. Follow-up

The follow-up endpoint was September 30, 2023, and the median follow-up time was 1613 (285–3910) days. The main methods of follow-up were telephone interviews, medical record inquiries, and efficacy evaluations combined with laboratory results. Patient OS, defined as the time from the beginning of treatment to death or the last follow-up, was analyzed.

### 2.5. Statistical analysis

SPSS 26.0 software (IBM Corp., Armonk) was used for statistical analysis of all the data. The chi-square test or Fisher exact test was used to compare data between groups. The Kaplan–Meier method and log-rank test were used for survival analysis and survival curve generation. Univariate and multivariate Cox regression models were used to analyze the prognostic risk factors, and *P* < .05 was considered statistically significant.

## 3. Results

### 3.1. Patient scale, clinical characteristics, and novel detection methods

The general clinical data of the 439 patients are summarized in Table 1.

**Table 1:** NDMM general data characteristics.

Total (N = 439)	
Gender, n (%)	
Male	245 (55.81)
Female	194 (44.19)
Age, median (R)	64 (33–91)
30–45 years old	9 (2.05)
46–60 years old	154 (35.08)
61–75 years old	215 (48.97)
>76 years old	61 (13.89)
M-protein type, n (%)	
IgG	204 (46.47)
IgGλ	95 (21.64)
IgGκ	109 (24.83)
IgA	109 (24.83)
IgAλ	59 (13.44)
IgAκ	50 (11.39)
IgM	1 (0.23)
IgMκ	1 (0.23)
IgD	11 (2.51)
IgDλ	9 (2.05)
IgDκ	2 (0.46)
IgE	2 (0.46)
IgEλ	1 (0.23)
IgEκ	1 (0.23)
Light chain	81 (18.45)
Light chainλ	33 (7.52)
Light chainκ	48 (10.93)
Missing value	31 (7.06)
DS stage, n (%)	
I	5 (1.14)
A	5 (1.14)
B	0 (0.00)
II	41 (9.34)
A	37 (8.43)
B	4 (0.91)
III	364 (82.91)
A	219 (49.89)
B	145 (33.03)
Missing value	26 (6.61)
ISS stage, n (%)	
I	56 (12.76)
II	90 (20.50)
III	250 (56.95)
Missing value	43 (9.79)
Extramedullary infiltration at initial diagnosis, n (%)	271 (61.73)
Yes	25 (9.22)
First symptom, n (%)	
Osteodynia	265 (60.36)
Anemia	128 (29.16)
Renal damage	42 (9.57)
Infection (cough, fever, etc)	39 (8.88)
Digestive system symptoms (nausea, vomiting)	40 (9.11)
Respiratory symptom (chest tightness, tightness, etc)	32 (7.29)
Nervous system symptoms (numbness and pain of both lower limbs, etc)	20 (4.56)
Combined with more than 2 kinds of combined symptoms	113 (25.74)
First diagnosis department, n (%)	247 (56.26)
Department of hematopathology	
Nephrology department	44 (10.02)
Pain department	31 (7.06)
Orthopedics department	31 (7.06)
Pneumology department	9 (2.05)
Neurology department	9 (2.05)
Other departments	68 (15.49)

DS = Durie–Salmon, NDMM = newly diagnosed multiple myeloma.

The clinical data of the 439 patients who underwent auxiliary examinations are summarized in Table 2.

**Table 2:** General laboratory examination characteristics.

Total (N = 439)	
Hemanalysis	
WBC, n (%)	
≥4 × 10^9^/L	334 (76.08)
<4 × 10^9^/L	105 (23.91)
Data missing	10 (2.28)
Hb, n (%)	
<85 g/L	293 (66.74)
85 ≤ Hb ≤ 100 g/L	77 (17.54)
>100 g/L	64 (14.58)
Data missing	5 (1.14)
PLT, n (%)	
<125 × 10^9^/L	129 (29.38)
≥125 × 10^9^/L	297 (67.65)
Data missing	13 (2.96)
CRP, n (%)	
>8 mg/L	58 (13.21)
≤8 mg/L	226 (51.48)
Data missing	155 (35.30)
Blood biochemistry LDH, n (%)	
>245 U/L	85 (19.36)
≤245 U/L	249 (56.72)
Data missing	105 (23.92)
Cr, n (%)	
>177 µmol/L	235 (29.16)
≤177 µmol/L	282 (64.23)
Data missing	29 (6.61)
UA, n (%)	
>430 µmol/L	235 (53.53)
≤430 µmol/L	169 (38.50)
Data missing	35 (7.97)
BUN, n (%)	
>8.3 mmol/L	156 (35.53)
≤8.3 mmol/L	241 (54.90)
Data missing	42 (9.57)
Ca, n (%)	
>2.65 mmol/L	70 (15.94)
≤2.65 mmol/L	327 (74.49)
Data missing	42 (9.57)
β_2_-MG, n (%)	
>5.5 mg/L	249 (56.72)
≤5.5 mg/L	149 (33.94)
Data missing	41 (9.33)
ALB, n (%)	
<35 g/L	308 (70.16)
≥35 g/L	108 (24.60)
>55 g/L	2 (0.46)
Data missing	23 (5.24)

**Figure 1.**
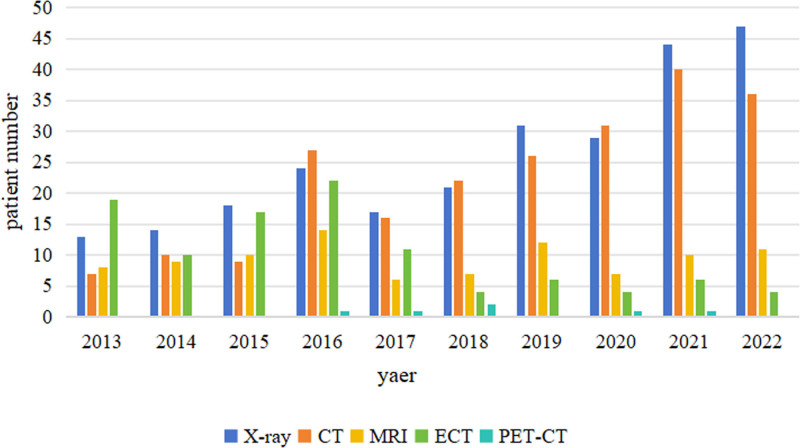
NDMM imaging examination from 2013 to 2022. NDMM = newly diagnosed multiple myeloma.

**Figure 2.**
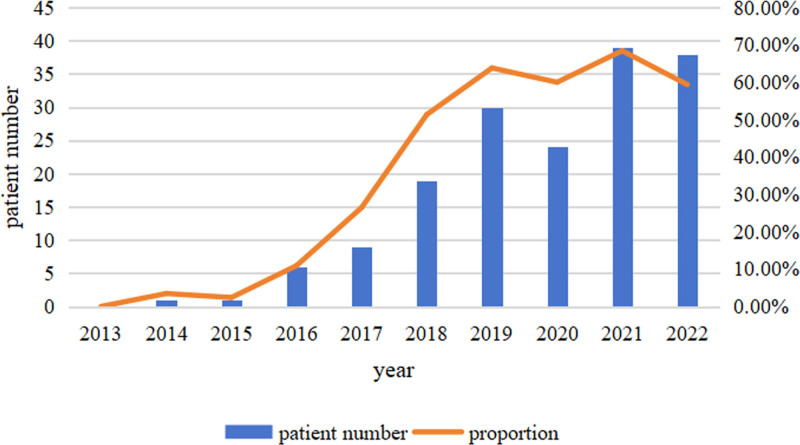
FISH usage of NDMM from 2013 to 2022. NDMM = newly diagnosed multiple myeloma.

Medical centers have also undergone tremendous changes in the application of new detection methods (Fig. 1). Only 271 patients (61.73%) underwent magnetic resonance imaging, CT or positron emission tomography/CT examinations, as shown in Table 3. A total of 168 patients (38.27%) underwent FISH examinations, and the percentage of patients undergoing this method tended to increase annually (from 0% in 2012–2015 to 68.42% in 2021) (Fig. 2).

**Table 3:** Other inspection features.

Total (N = 439)	
BMPCs, n (%)	
>60%	53 (12.07)
≤60%	376 (85.65)
Data missing	10 (2.28)
Iconography, n (%)	
X-rays	258 (58.77)
ECT	103 (23.46)
CT	224 (51.03)
MRI	95 (21.64)
PET-CT	6 (1.37)
Imaging abnormality, n (%)	373 (84.97)
Data missing, n (%)	26 (5.92)
Bone destruction, n (%)	
Osteolytic destruction	232 (52.85)
Pathological fracture	85 (19.36)
Osteoporosis	174 (39.63)
Nothing abnormal detected	40 (9.11)
FISH, n (%)	168 (38.27)
Positive	115 (68.45)
Negative	71 (31.55)
RB1 deletion	61 (53.04)
D13S319 deletion	36 (31.30)
IGH rearrangement	48 (41.74)
p53 gene deletion	22 (19.13)
1q21 amplification	81 (70.43)
Single genetic abnormality	44 (26.19)
≥2 genetic abnormalities	71 (42.26)

BMPC = bone marrow progenitor cells, CT = computed tomography, MRI = magnetic resonance imaging, PET = positron emission tomography.

### 3.2. Treatment conditions

**Figure 3.**
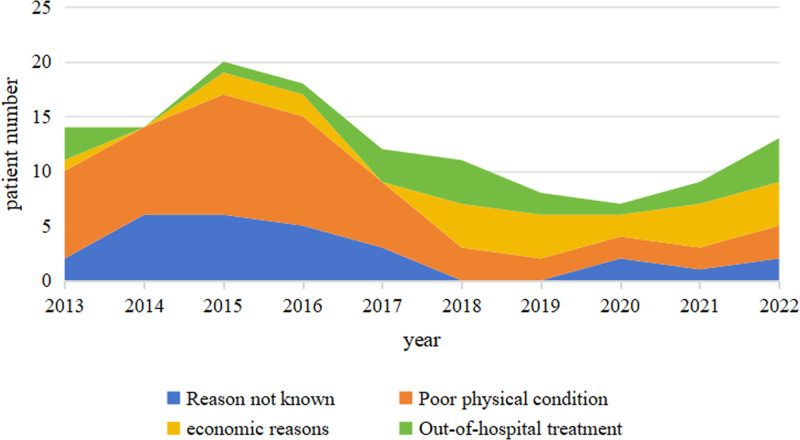
Distribution of NDMM abandonment treatment from 2013 to 2022. NDMM = newly diagnosed multiple myeloma.

**Figure 4.**
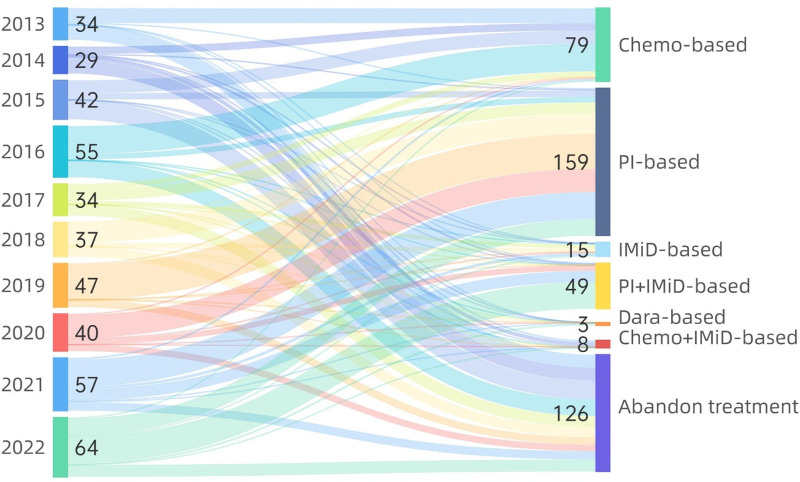
The number of patients receiving induction therapy and the distribution of chemotherapy regimens.

The treatment methods of the 439 patients with NDMM is shown in Table 4. A total of 126 patients (28.70%) withdrew from treatment in our hospital immediately after diagnosis (Fig. 3). Among them, 55 patients (43.65%) could not be treated with chemotherapy due to poor physical condition, 23 patients (18.25%) refused chemotherapy for economic reasons, 21 patients (16.67%) received further chemotherapy in other hospitals, and 27 patients (21.43%) were lost to follow-up. Furthermore, 313 patients (71.30%) received induction therapy, including 79 patients (25.24%) who received traditional chemotherapy, 8 patients (2.56%) who received chemotherapy + IMiDs, 15 patients (4.79%) who received IMiD-based therapy, 159 patients (50.80%) who received PI-based therapy, 49 patients (15.65%) who received PI + IMiD-based therapy, and 3 patients (0.96%) who received CD38 monoclonal antibody-based therapy. Induction therapy for NDMM patients has undergone tremendous changes in recent years (Fig. 4). From 2013 to 2016, the main induction therapy for NDMM patients was traditional chemotherapy, and only some patients received IMiDs in combination with traditional chemotherapy. From 2017 to 2019, the main induction treatment was a PI-based regimen, with some patients using an IMiD-based regimen. From 2020 to 2022, the main induction therapies were PI-based or PI + IMiD-based regimens, and the proportion of patients receiving PIs + IMiDs gradually increased.

**Table 4:** Treatment of MM.

Total (N = 439)		
Abandon treatment, n (%)		126 (28.7)
	Physical reason	55 (43.65)
	Economic reason	23 (18.25)
	Further chemotherapy to other hospitals	21 (16.67)
	Missing visit	27 (21.43)
Inductive treatment, n (%)		313 (71.30)
	Chemo-only	79 (25.24)
	Chemo + IMiD-based	8 (2.56)
	IMiD-based	15 (4.79)
	PI-based	159 (50.80)
	IMiD + PI-based	49 (15.65)
	Ddaratumumab-based	3 (0.96)
Early ASCT, n (%)		23 (7.35)
	PD (cutoff observation end point)	5 (21.74)
	CR (cutoff observation end point)	18 (78.26)
Maintenance treatment, n (%)		108 (34.50)
	Chemo-only	12 (11.11)
	IMiD-based	56 (51.85)
	PI-based	23 (21.30)
	PI + IMiD-based	15 (13.89)
	Ddaratumumab-based	2 (1.85)
Without maintenance treatmen, n (%)		205 (65.49)
	Refusal of treatment or short-term death	177 (56.55)
	Disease progression or recurrence	28 (8.95)
Progress to RRMM, n (%)		105 (33.55)
	Continuous treatment	97 (92.38)
	CAR-T	6 (6.18)
	Salvage ASCT	4 (3.81)
	Abandon treatment	8 (7.62)

ASCT = autologous stem cell transplantation, CAR = chimeric antigen receptor, CR = complete remission, IMiDs = immunomodulatory drugs, MM = multiple myeloma, PD = disease progression, PIs = proteasome inhibitors, RRMM = refractory multiple myeloma.

**Figure 5.**
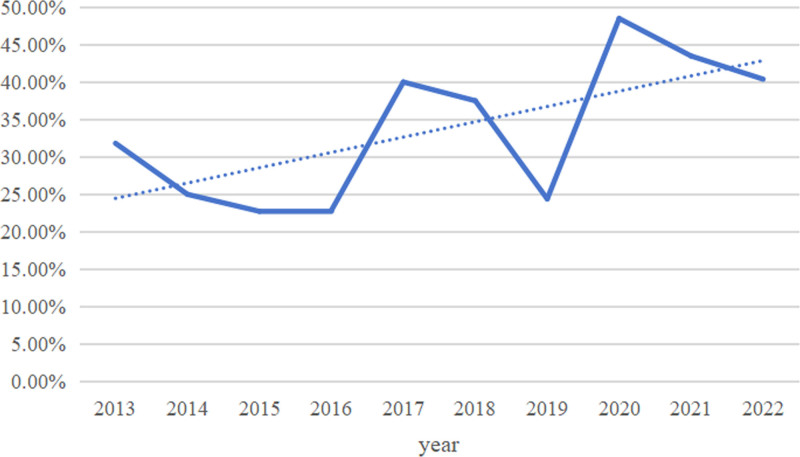
Maintenance treatment of NDMM patients from 2013 to 2022. NDMM = newly diagnosed multiple myeloma.

**Figure 6.**
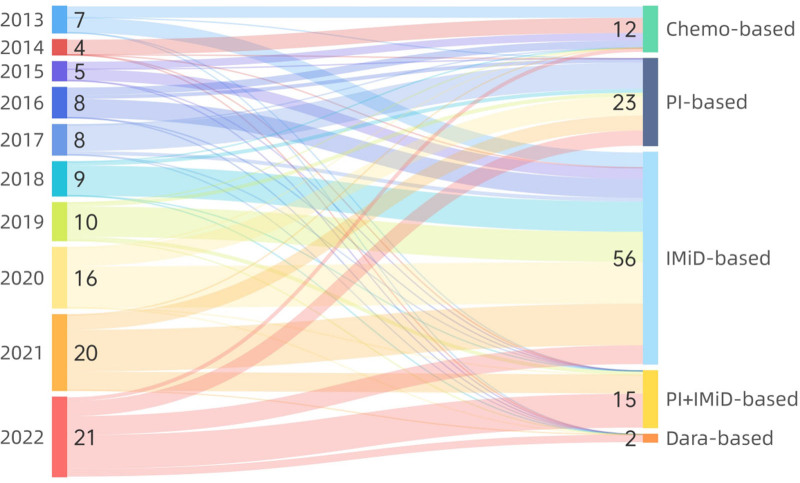
The number of patients receiving maintenance treatment and the distribution of chemotherapy regimens.

Among the 313 patients who received induction therapy, 23 (7.35%) patients underwent early ASCT after achieving at least PR after induction therapy. Among them, 5 (21.74%) patients experienced recurrence, and 18 (78.26%) patients achieved CR that was maintained at the end of the observation period (September 30, 2023). A total of 108/313 patients entered maintenance treatment by the end of follow-up (September 30, 2023), including 12 patients (11.11%) who used chemotherapy, 23 patients (21.30%) who used PIs, 56 patients (51.85%) who used IMiDs, 15 patients (13.89%) who used PIs + IMiDs, and 2 patients (1.85%) who used CD38 monoclonal antibodies. The proportion of patients who received maintenance therapy increased over time (Fig. 5), which was positively correlated with the annual improvement in the remission rate after 1st-line induction therapy. The main chemotherapy regimens changed from traditional chemotherapy to PI-based and/or IMiD-based double or triple chemotherapy. The changing trends of maintenance therapy for NDMM patients in different years are shown in Figure 6. In total, 205 (65.49%) patients did not enter maintenance treatment; of these, 177 (56.55%) patients either refused or died during induction treatment, and 28 (8.95%) patients failed to respond to the induction consolidation phase.

A total of 105 patients (33.55%) progressed or relapsed during follow-up. Ninety-seven patients (92.38%) underwent next-line treatment; among these, 6 patients (6.18%) underwent chimeric antigen receptor (CAR)–T-cell therapy, 4 patients (3.81%) underwent salvage ASCT after multiline recurrence, and 8 patients (7.62%) either discontinued treatment or died.

### 3.3. Treatment response

Among the 313 patients who received induction therapy, 82 (26.20%) patients had no accurate response result by the end of follow-up. Among them, 60 (19.17%) patients discontinued treatment after receiving 1 to 2 courses, and 22 (7.03%) patients could not receive follow-up chemotherapy and died. A total of 231 (73.80%) NDMM patients had a clear response to 1st-line induction therapy (Table 5). Among them, the proportions of patients who achieved CR, VGPR, PR, stable disease, and disease progression were 20.35%, 22.51%, 29.87%, 9.09%, and 18.18%, respectively. Approximately 72.73% of patients achieved disease remission, and 42.86% of patients achieved deep remission. The median number of treatment courses was 4.

**Table 5:** Remission rate of NDMM 1st-line induction therapy.

	Total (N = 231)
Best confirmed response, n (%)	
ORR	168 (72.73)
CR	47 (20.35)
VGPR	52 (22.51)
PR	69 (29.87)
SD	21 (9.09)
PD	42 (18.18)
Cycles, median (range)	4

CR = complete remission, NDMM = newly diagnosed multiple myeloma, ORR = overall response rate, PD = disease progression, PR = partial remission, SD = stable disease, VGPR = very good partial remission.

The 1st-line induction therapy mode varied greatly over time. The study sample was divided into 3 groups according to treatment year (2013–2016, 2017–2019, and 2020–2022), and the differences in treatment modes were assessed. There was a statistically significant difference in the remission rate after 1st-line induction therapy among the 3 groups (χ^2^ = 20.040, *P* < .001) (Table 6). The differences in the remission rate after 1st-line induction therapy between the groups in each year were evaluated using the Bonferroni method, and the test level α was redefined such that *P* < .0166 was statistically significant. The results were as follows: first, there was no difference in the disease remission rate between the 2013 to 2016 group and the 2016 to 2019 group. Second, the remission rate in the 2020 to 2022 group was better remission rate than that in the 2013 to 2016 group. Third, the remission rate in the 2020 to 2022 group was also better than that in the 2017 to 2019 group (Table 7).

**Table 6:** Comparison of disease remission rates of NDMM in 2013 to 2016, 2017 to 2019, and 2020 to 2022.

Comparative group	Number of cases	CR (%)	VGPR (%)	Disease remission	Disease not alleviated	χ^2^	*P*
2013–2016 yr	65	12 (18.46)	10 (15.38)	35 (53.85)	30 (46.15)	20.040	<.001
2017–2019 yr	62	10 (16.13)	13 (20.97)	43 (69.35)	19 (30.64)		
2020–2022 yr	104	25 (24.04)	29 (27.88)	90 (86.54)	14 (13.46)		

*Note*: The efficacy evaluation was CR, VGPR, and PR for disease remission, while the efficacy evaluation was SD and PD for disease no remission.

CR = complete remission, NDMM = newly diagnosed multiple myeloma, PD = disease progression, PR = partial remission, SD = stable disease, VGPR = very good partial remission.

**Table 7:** Comparison of disease remission rates between groups in 2013 to 2016, 2017 to 2019, and 2020 to 2022.

Comparative group	Disease remission	Disease not alleviated	Total	χ^2^	*P*
2013–2016 yr	35	30	65	3.221	.073
2017–2019 yr	43	19	62		
Total	78	49	127		
2013–2016 yr	35	30	65	22.200	<.001
2020–2022 yr	90	14	104		
Total	125	44	169		
2017–2019 yr	43	19	62	7.201	.007
2020–2022 yr	90	14	104		
Total	133	33	166		

*Note*: *P* < .016 was statistically significant.

The efficacy of 1st-line induction therapy in NDMM patients with different ages, International Staging System (ISS) stages, and induction regimens was also compared (Table 8). There was a statistically significant difference in efficacy between different groups of patients (*P* < .05). The Bonferroni method was used to further compare the different induction treatment groups (Table 9), and the test level α was redefined such that *P* < .0166 was considered statistically significant. In addition, the efficacy of chemotherapy regimens with new drugs was better than that of traditional chemotherapy regiments (*P* < .016).

**Table 8:** Analysis of 1st-line induction efficacy in different groups of NDMM patients.

Comparative group	Disease remission	Disease not alleviated	Total	χ^2^	*P*
<64 yr	102	24	126	8.464	.004
≥64 yr	61	35	96		
Total	59	163	222		
ISS stage I/II	77	18	95	4.953	.026
ISS stage III	86	41	127		
Total	163	59	222		
Chemo-based	28	24	52	16.047	<.001
PI/IMiD-based	100	35	135		
PI + IMiD-based	38	4	42		
Total	166	63	229		

*Note*: *P* < .05 was statistically significant.

IMiDs = immunomodulatory drugs, ISS = International Staging System, NDMM = newly diagnosed multiple myeloma, PIs = proteasome inhibitors.

**Table 9:** Efficacy analysis of different induction therapy regimens.

Comparative group	Disease remission	Disease not alleviated	Total	χ^2^	*P*
Chemo-based	28	24	52	7.112	.008
PI/IMiD-based	100	35	135		
Total	128	59	187		
Chemo-based	28	24	52	14.906	<.001
PI + IMiD-based	38	4	42		
Total	28	66	94		
PI/IMiD-based	100	35	136	5.017	.025
PI + IMiD-based	38	4	42		
Total	138	39	177		

*Note*: *P* < .016 was statistically significant.

IMiDs = immunomodulatory drugs, PIs = proteasome inhibitors.

### 3.4. Improvements in patient survival

**Figure 7.**
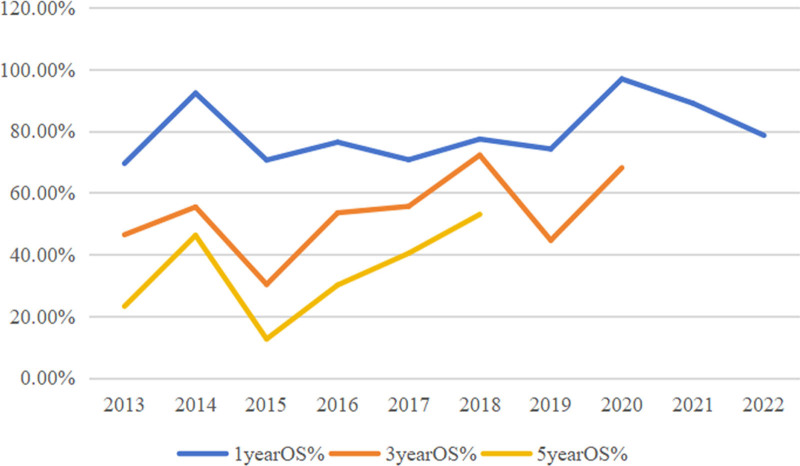
Survival rate changes from 2013 to 2022.

A total of 313 NDMM patients who received induction therapy were followed up to September 30, 2023. The median follow-up time was 1613 (285–3910) days. A total of 260 patients completed the follow-up period, with a follow-up rate of 83.07%. Among them, 116 (37.06%) patients survived, and 144 (46.01%) died; the other 53 (16.93%) patients were lost to follow-up. The median OS was 1273 days, and the 1-, 3-, and 5-year OS rates from 2013 to 2022 were 80.50%, 55.50%, and 35.70%, respectively. In this period, the 1-, 3-, and 5-year OS rates of patients tended to increase annually (Fig. 7), and the OS of patients after 2020 improved significantly.

#### 3.4.1. OS and survival curves

**Figure 8.**
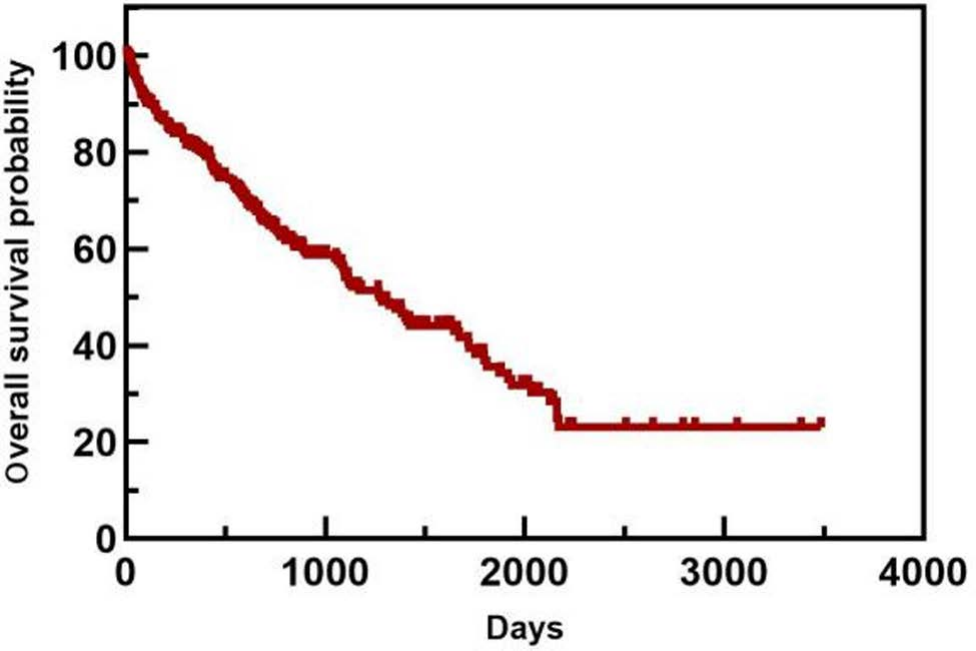
NDMM overall OS. NDMM = newly diagnosed multiple myeloma, OS = overall survival.

The OS curve (Fig. 8) and the survival curve of each group were plotted via the Kaplan–Meier method, and comparisons between groups were performed using the log-rank method (Table 10).

**Table 10:** The mOS of NDMM patients in different groups.

Variate	n (number)	mOS (d)	95% CI	χ^2^	*df*	*P*
Age	313	1273	1060.608–1485.392	27.218	1	<.001***
<64 yr	160	1813	1618.408–2007.592			
≥64 yr	153	743	535.567–950.433			
Sex	313	1273	1060.608–1485.392	0.167	1	.683
Male	168	1268	950.393–1585.607			
Female	145	1322	863.070–1780.930			
DS	310	1285	1070.076–1499.924	0.013	1	.909
I/II	37	1380	1006.960–1753.040			
III	273	1273	1032.039–1513.961			
ISS	297	1780	1011.331–1748.669	8.960	1	.003**
I/II	119	1813	1329.824–2296.176			
III	178	1089	791.190–1386.810			
ASCT or not	313	1273	1060.608–1485.392	13.915	1	<.001***
ASCT	27	–	–			
No ASCT	286	1117	844.890–1389.110			
Background disease	313	1273	1060.608–1485.392	0.290	1	.590
Yes	256	1268	1015.301–1520.699			
No	57	1380	986.714–1773.286			

ASCT = autologous stem cell transplantation, DS = Durie–Salmon, ISS = International Staging System, mOS = median OS, NDMM = newly diagnosed multiple myeloma.

* *P* < .05.

** *P* < .01.

*** *P* < .001.

**Figure 9.**
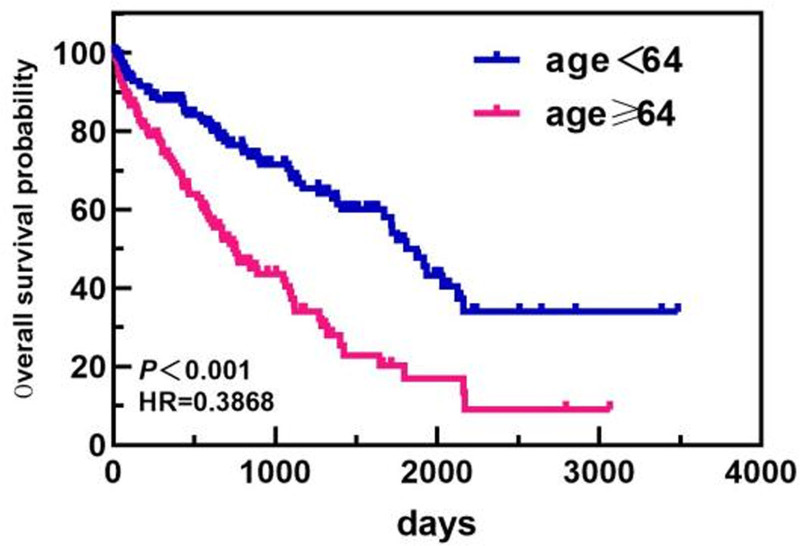
Comparison of OS in patients of different ages. OS = overall survival.

**Figure 10.**
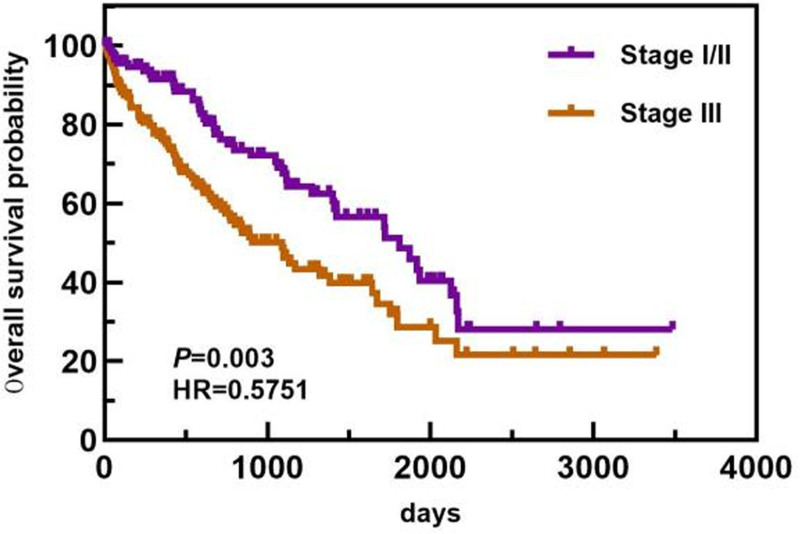
Comparison of OS in patients with different ISS stages. ISS = International Staging System, OS = overall survival.

**Figure 11.**
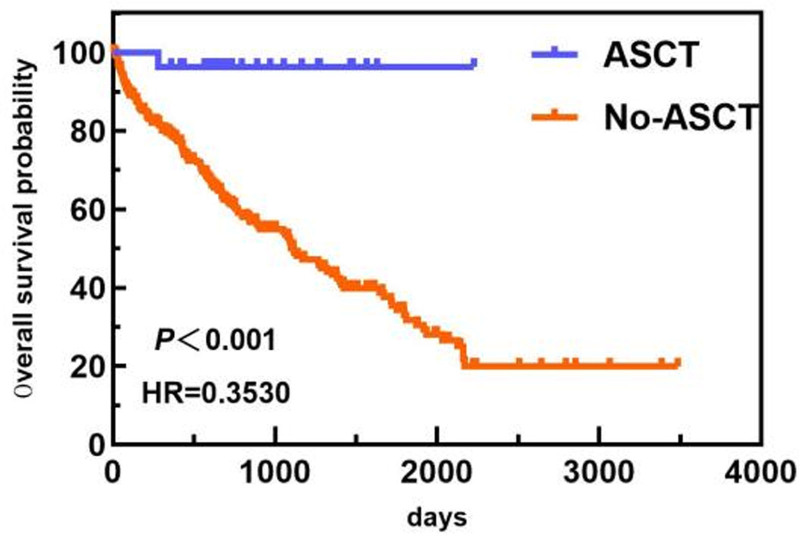
Comparison of OS between ASCT patients and non-ASCT patients. ASCT = autologous stem cell transplantation, OS = overall survival.

**Figure 12.**
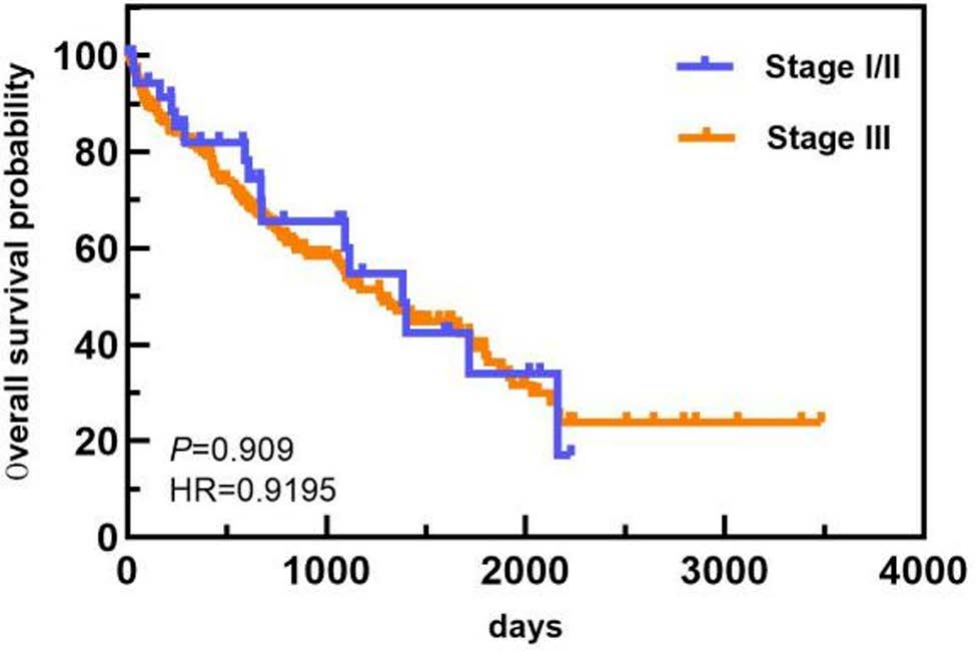
Comparison of OS in patients with different DS stages. DS = Durie–Salmon, OS = overall survival.

**Figure 13.**
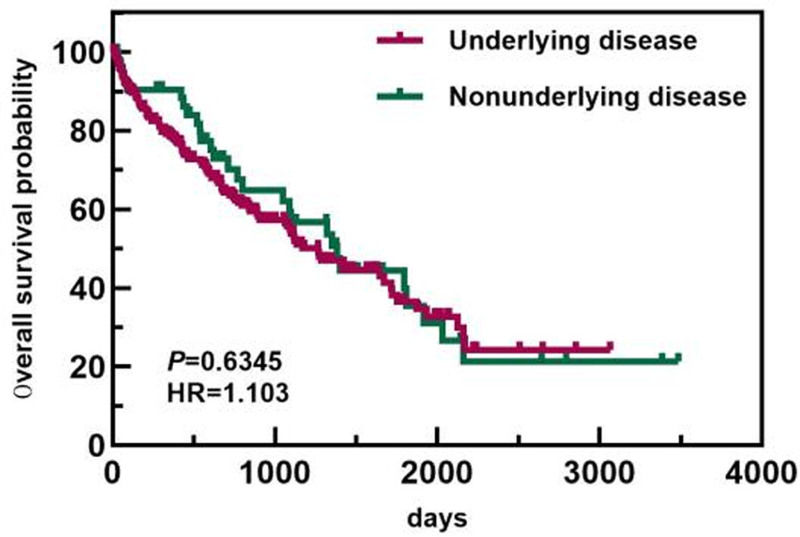
Comparison of OS between patients with and without underlying diseases. OS = overall survival.

**Figure 14.**
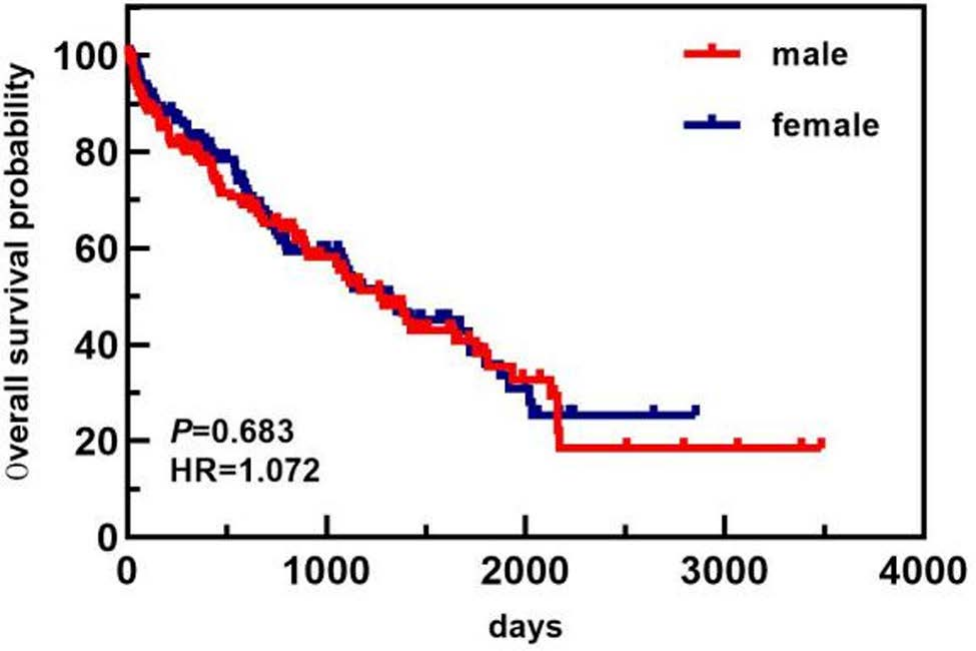
Comparison of OS in patients of different genders. OS = overall survival.

In the age subgroup, patients <64 years old had a longer OS than patients ≥64 years old did (1813 days vs 743 days). In the ISS staging subgroup, the OS of patients with ISS stage I/II disease was greater than that of patients with ISS stage III disease (1813 days vs 1089 days). In the ASCT subgroup, the median OS of patients who did not undergo ASCT was 1117 days. Only 1 patient died in the ASCT group, and the median OS was not calculated. The OS of patients who underwent ASCT was greater than that of patients who did not undergo ASCT. The differences in median OS between these groups were statistically significant (*P* < .05). The overall and subgroup survival curves are shown in Figures 9–11. Among the different sexes, the median OS of women was greater than that of men (1322 days vs 1268 days). In the Durie–Salmon stage subgroup, the median OS in patients with stage I/II disease was greater than that in patients with stage III disease (1380 days vs 1273 days). In the basic disease subgroup, the median OS of people without a history of basic diseases was greater than that of people with basic diseases (1380 days vs 1268 days). The differences in median OS between these groups were not statistically significant (*P* > .05). The survival curve of each group is shown in Figures 12–14.

#### 3.4.2. Prognostic factors

Univariate analysis was conducted to study the prognostic factors for OS; age >65 years, ISS stage III, PLT count <100 × 10^9^/L, β_2_-MG ≥ 5.5 mg/L, increased LDH levels, bone marrow progenitor cells ≥ 60%, uric acid (UA) ≥ 430 μmol/L, Ca ≥ 2.65 mmol/L, progression to relapsed/refractory multiple myeloma (RRMM), and no ASCT were all found to be adverse prognostic factors affecting OS, with *P* < .05 (Table 11). The factors with *P* < .05 in the univariate analysis were included in the multivariate Cox regression model. The results revealed that age >65 years, ISS stage III, no ASCT, β_2_-MG ≥ 5.5 mg/L, progression to RRMM, UA ≥ 430 μmol/L, and LDH ≥ 245 U/L were independent adverse prognostic factors affecting OS (*P* < .05) (Table 12).

**Table 11:** Univariate COX regression analysis.

	HR (95% CI)	*P*
OS		
Age > 65 yr	0.486 (0.344–0.688)	<.001***
Sex (male)	1.072 (0.767–1.498)	.683
IgG	0.863 (0.616–1.210)	.393
IgA	1.216 (0.814–1.816)	.340
Light chain	1.112 (0.720–1.716)	.633
ISS stage Ⅲ	0.573 (0.396–0.829)	.003**
DS stage Ⅲ	0.971 (0.584–1.614)	.909
ASCT (no)	15.957 (2.231–114.145)	.006**
RRMM (yes)	1.748 (1.212–2.521)	.003**
WBC ≥ 4 × 10^9^/L	0.903 (0.604–1.350)	.619
Hb ≤ 100 g/L	0.644 (0.377–1.103)	.109
PLT ≤ 100 × 10^9^/L	0.613 (0.380–0.990)	.045*
β_2_-MG>5.5 mg/L	0.625 (0.435–0.898)	.011*
ALB < 40 g/L	1.212 (0.736–1.994)	.450
Cr ≥ 177 µmol/L	0.705 (0.486–1.023)	.066
UA ≥ 430 µmol/L	0.620 (0.390–0.986)	.044*
Ca ≥ 2.65 mmol/L	1.854 (1.299–2.645)	.001***
LDH ≥ 245 U/L	0.505 (0.326–0.782)	.002**
1q21 amplification	1.454 (0.934–2.263)	.097
D13S319 deletion	1.272 (0.731–2.212)	.395
IGH rearrangement	1.377 (0.805–2.354)	.242
p53 gene deletion	1.296 (0.570–2.944)	.536
RB1 deletion	1.373 (0.854–2.207)	.191
BMPCs ≥ 60%	0.577 (0.371–0.899)	.015*
Underlying diseases (yes)	0.892 (0.588–1.353)	.590

ASCT = autologous stem cell transplantation, BMPC = bone marrow progenitor cells, DS = Durie–Salmon, ISS = International Staging System, LDH = lactate dehydrogenase, OS = overall survival, RRMM = refractory multiple myeloma, UA = uric acid, β2-MG = β2-microglobulin.

* *P* < .05.

** *P* < .01.

*** *P* < .001.

**Table 12:** Multivariate COX regression analysis.

	HR (95% CI)	*P*
OS		
Age > 65 yr	0.585 (0.369–0.926)	.022*
ISS stageⅢ	0.072 (0.008–0.641)	.018*
β_2_-MG > 5.5 mg/L	11.078 (1.283–95.650)	.029*
RRMM (yes)	1.703 (1.073–2.705)	.024*
ASCT (no)	10.328 (1.427–74.744)	.021*
UA ≥ 430 µmol/L	0.639 (0.411–0.995)	.047*
LDH ≥ 245 U/L	0.573 (0.356–0.920)	.021*

ASCT = autologous stem cell transplantation, ISS = International Staging System, LDH = lactate dehydrogenase, OS = overall survival, RRMM = refractory multiple myeloma, UA = uric acid, β2-MG = β2-microglobulin.

* *P* < .05.

## 4. Discussion

In the past 10 years, the economy, medical care, education, and other aspects of the Gannan region in Jiangxi Province, China, have significantly improved. The per capita life expectancy of the population in this region has increased, and compliance has significantly improved, as have the number of NDMM diagnoses and follow-up treatments. In China, provinces with a higher per capita GDP bear a greater burden of MM, a pattern that parallels observations in Western developed countries.^[11,15,16]^ The male-to-female ratio of NDMM patients in this study was 1.26:1, and the median age was 64 years (33–91 years). Among them, 154 patients (35.08%) were 46 to 60 years old, and 215 patients (48.97%) were 61 to 75 years old. The peak age of onset in other countries is close to 66 years old,^[17]^ and the median age of 57 years (55–74 years). A significant increasing trend in the MM burden is evident among young adults (20–35 years) in China, a phenomenon compounded by a large epidemic peak in the patient population.^[10,11,18]^ The proportion of patients with underlying diseases in this study was 80.64%, and the proportion of patients with ISS stage III disease was 82.91%. In addition, the proportion of patients in the late ISS stage (29.7–48.3%) was significantly greater than that reported in previous studies.^[19–21]^

In resource-constrained countries and regions, patients are usually less willing to continue treatment for MM. As shown in Table 4, 29.16% of our patients discontinued treatment immediately after diagnosis. Follow-up by telephone interview and medical record inquiry revealed that from 2013 to 2017, most patients discontinued treatment due to poor physical status, which may be related to the late diagnosis of the disease and low awareness of health problems. From 2018 to 2022, the proportion of patients who discontinued treatment decreased significantly compared with the 2013 to 2017 group, indicating that patients’ willingness to seek medical treatment has increased significantly since 2018. The improvement in medical awareness and willingness to see a doctor has promoted early diagnosis of diseases and encouraged most patients to undergo active treatment; late-stage patients may also be more willing to use daratumumab-containing chemotherapy or to try or use IMiD-containing treatment regimens for palliative treatment, reducing the number of patients who discontinue treatment immediately after diagnosis for physical reasons. As shown in Figures 4, 71.30% (313/439) of NDMM patients received induction therapy, and the regimen changed substantially among the observed patients. From 2013 to 2016, the main induction therapy for NDMM patients was traditional chemotherapy (VAD, MP, etc), and only some patients used thalidomide or lenalidomide in addition to traditional chemotherapy. From 2017 to 2019, the main induction therapy was based on a PI (bortezomib), and a small number of patients used an IMiD (lenalidomide). From 2020 to 2022, the main induction therapies were based on PIs (bortezomib, ixazomib) and PIs + IMiDs (VRD, IRD, etc), and the proportion of patients receiving PIs + IMiDs gradually increased. A total of 7.35% (23/439) of patients underwent early ASCT. By the end of the observation period, 34.50% (108/313) of the patients had entered the maintenance treatment stage, and this proportion tended to increase annually. As shown in Figure 6, the maintenance chemotherapy regimen has shifted from traditional chemotherapy to double or triple chemotherapy with PIs or IMiDs. A total of 33.55% (105/313) of patients eventually progressed to RRMM, and 92.38% (97/105) of patients continued to receive treatment, of whom 6.18% received CAR–T-cell treatment and 3.81% underwent salvage ASCT after multiline recurrence. The proportion of patients receiving maintenance treatment increased over time but decreased after 2019. Considering the outbreak and epidemic of the new coronavirus in December 2019, the dynamic management of the epidemic situation in various provinces across the country led to a significant decline in patient compliance, and the increase in the mortality rate of COVID-19 meant that patients did not enter the maintenance stage in a timely manner. In 1st-tier cities, most patients began to use new drugs in 2015, and ASCT and other therapies were carried out earlier.^[9]^ Our medical center began to perform hematopoietic stem cell transplantation and CAR–T-cell immunotherapy in 2022. A number of new drugs were not incorporated into the national medical insurance until 2017, and the number of users increased significantly. Compared with 1st-tier cities in China, this incorporation was gradual and occurred 2 years later. Considering the economic status of residents, most people find it difficult to accept higher-priced chemotherapy drugs. Therefore, from 2017 to 2019, most patients were treated with PI-based induction therapy. As the VRD regimen was further integrated into national health insurance, more patients could receive a triple regimen, including PIs and IMiDs (most commonly VRD), for the period 2020 to 2022. The reasons for these changes include the improved accessibility to new drugs and the expansion of indications covered by the national medical insurance policy. For maintenance treatment, the changes in the treatment plan are related to the significant improvement in the remission rate of 1st-line induction treatments containing new drugs. The development of more new technologies and reports of their efficacy have increased patient compliance and their willingness to continue follow-up maintenance treatment and post-recurrence treatment.

In most domestic studies, the use of ASCT in China has shown a continuous growth trend.[^[22–24]^ However, the current proportion of Chinese patients receiving 1st-line ASCT is only 12.6% to 14.4%,^[20]^ far less than that in Western countries; a realistic study in Australia and New Zealand^[25]^ reported that the utilization rate of 1st-line ASCT was 67%, and the overall utilization rate was 76%. In a realistic study in Finland, the utilization rate of ASCT was 30%, and the utilization rate of patients under 65 years of age was 60.5%.^[26]^ Many domestic and foreign studies have shown that ASCT can improve the progression-free survival (PFS) and OS of patients.^[27–29]^ Among these studies, the NICHE-MM sub-cohort of a continuous, longitudinal, national, multi-disease cohort study of hematological patients (NICHE study) in China was evaluated; PFS (42.9 months vs 21.2 months, *P* < .001) and OS (not reached vs 65.8 months, *P* < .001) of patients receiving ASCT as a 1st-line treatment were longer than those of patients not receiving ASCT. However, only 8.62% (27/313) of the patients in this study were treated with ASCT, including 23 patients who underwent early ASCT and 4 patients who underwent salvage ASCT. By the end of the observation period, 5 patients had experienced recurrence, and 18 patients achieved CR. Compared with those in patients without ASCT, the overall response rate, deep remission rate, and median OS were greater in the ASCT group. The prognostic analysis in this study also revealed that ASCT had a positive effect on OS prolongation. The proportion of ASCT patients was much lower than that reported in foreign studies and was also lower than that reported in most domestic studies. The reason for this difference may be that the ASCT technology in our medical center has a short development time, the transplantation level needs to be further improved, and the treatment cost is high. In the current medical environment, new subcutaneous or oral drug regimens are easy to use, and the price is relatively acceptable. Owing to patients’ views, economic ability, and concerns about side effects, more patients hesitate to undergo transplantation and prefer non-transplantation. With the continuous progress of technology in this medical center, preferential medical insurance policies, and vigorous popularization and publicity of ASCT technology, the utilization rate of subsequent ASCT may be further improved.

As shown in Table 5, the remission rate after 1st-line induction therapy in this medical center differs substantially from those in related studies at home and abroad. As shown in Tables 8 and 9, age, ISS stage, and induction therapy with new drugs can all increase the efficacy of 1st-line induction therapy. A retrospective study of Zhongshan Hospital, Fudan University, Shanghai, from 2007 to 2021 revealed that 55.2%, 10.6%, 27.0%, and 7.0% of patients received 1st-line treatment with PI-based, IMiD-based, PI + IMiD-based, and traditional chemotherapy, respectively. A total of 86.5% of patients achieved disease remission, and 62.6% of patients achieved deep remission.^[9]^ In the study by Chen Juan et al, 57.5%, 18.7%, 16.4%, and 7.4% of the patients received PI-based, IMiD-based, PI + IMiD-based, and traditional chemotherapy-based 1st-line treatment, respectively. After 4 courses of treatment, the efficacy was evaluated; 70.9% of the patients achieved disease remission, and 38.1% of the patients achieved deep remission.^[30]^ A retrospective multicenter study of 454 NDMM patients in China revealed that the total effective rates of the PI-based, IMiD-based, PI + IMiD-based, and conventional regimens were 91.0%, 83.9%, 90.6%, and 60.9%, respectively, and were significantly related to their superior prognoses.^[29]^ A retrospective study in Latin America revealed that the main induction regimens used were an alkylation regimen (18.2%), thalidomide regimen (67.8%), and bortezomib regimen (13.1%). The median OS (mOS) was 44 months, 20.1% of the patients achieved CR or better, and 44.9% of the patients achieved PR or VGPR. The effects of age, chemotherapy containing new drugs, and ASCT on mOS were analyzed (*P* < .05).^[22]^ This result may be related to the low overall economic status and medical level in the Gannan region, the difficulty in achieving accurate assessment and diagnosis, the lack of standardized treatment, poor patient compliance, and the small sample size. In the future, health education and medical guidance for patients should be strengthened to increase the enthusiasm of patients for diagnosis and treatment. With the gradual increase in the OS rate, the prognosis of patients has greatly improved, which indicates not only the substantial potential of new drugs and ASCT but also the important reference value of advanced detection technology and complete baseline examination in disease treatment.

In this study, 313 patients who received induction therapy were followed up for a median follow-up time of 1613 (285–3910) days. A total of 260 patients completed the follow-up period, with a follow-up rate of 83.07%. Among them, 116 patients (37.06%) survived, and 144 (46.01%) died; the remaining 53 patients (16.93%) were lost to follow-up. The mOS was 1273 days, the 3-year OS rate was 55.50%, and the 5-year OS rate was 35.70%. Furthermore, age, ISS stage and ASCT affected the OS of patients. According to CONCORD-3 2018 statistics, the overall 5-year survival rate of MM patients in China is 24.8%, which is still significantly lower than the proportions reported in the United States (46.7%) and other Asian countries (e.g., 33.3% in Japan).^[23]^ A retrospective study at Beijing Chaoyang Hospital revealed that the median follow-up time of all patients was 20 months, the estimated median PFS was 37.6 months, and the estimated mOS was 61.0 months. Age, cytogenetic status, and renal insufficiency are believed to affect patient prognosis, and ASCT and bortezomib-based regimens are believed to prolong PFS and OS.^[24]^ The SEER database reported a 5-year OS rate (57.9%) for 2012 to 2018. A retrospective study of patients at Zhongshan Hospital Affiliated with Shanghai Fudan University from 2007 to 2021 reported that the mOS was 64.7 months, and the 1-year OS rates from 2007 to 2012, 2015 to 2019, and 2020 to 2021 were 79.6%, 85.6%, and 93.1%, respectively. The 3-year OS rates before and after 2015 were 54.0% and 70.0%, respectively, and the 5-year OS rates before and after 2015 were 39.1% and 59.1%, respectively. Different ISS stages, cytogenetic status, and induction chemotherapy regimens affected mOS (*P* < .001).^[9]^ A national retrospective study from 2009 to 2013 in Finland reported that the median survival time of MM patients was 3.7 years, and the median survival time of patients diagnosed before 65 years of age was 6.7 years.^[26]^ A retrospective study at the Mayo Clinic from 2004 to 2015 revealed that the median follow-up time of NDMM patients was 6.2 years, the overall mOS was 7.5 years, and the 5-year survival rate was as high as 75%.^[31]^ In summary, the effects of age, ISS stage, and ASCT on the OS of patients in this study are consistent with those of the above studies. The overall mOS and survival rates in this study were greater than the overall mOS and survival rates in China but far lower than the overall mOS and survival rates of patients from Beijing Chaoyang Hospital from 2006 to 2011 and Zhongshan Hospital Affiliated with Shanghai Fudan University from 2007 to 2021, which were far lower than those of the Mayo Clinic from 2004 to 2015, even considering the significant time difference. This difference may be related to the small sample size or differences in follow-up cycles, economic and medical levels across different regions, standardized diagnosis and treatment levels, treatment options, and relevant policy support. Moreover, the management of MM is challenging in countries and regions with limited resources; thus, improving the accessibility of early accurate diagnosis and treatment is crucial for improving the survival outcomes of MM patients.

Our study revealed that age > 65 years, ISS stage III, PLT count < 100 × 10^9^/L, β_2_-MG > 5.5 mg/L, increased LDH levels, bone marrow progenitor cells ≥ 60%, UA ≥ 430 µmol/L, Ca > 2.65 mmol/L, progression to RRMM, and no ASCT were adverse prognostic factors affecting OS (all *P* < .05), whereas age ≥ 64 years, ISS stage III, no ASCT, β_2_-MG > 5.5 mg/L, progression to RRMM, UA ≥ 430 μmol/L, and LDH ≥ 245 U/L were independent prognostic factors affecting OS (*P* < .05). The preliminary results of an EMN02/H095 randomized trial of 1308 NDMM patients using multivariate Cox regression analysis revealed that, compared with new drugs, the PFS and OS rates of pre-ASCT patients were both greater.^[32]^ A retrospective analysis by Abdullah S Al Saleh et al revealed that thrombocytopenia (PLT count < 150 × 10^9^/L) and age > 64 years were independent risk factors for shorter PFS and OS (*P* < .001).^[13]^ According to a study by Yuqi Chen et al, high β_2_-MG, high serum LDH, and low serum albumin levels are poor prognostic factors for early death.^[33]^ A multicenter retrospective study in China included 454 patients with NDMM, and multivariate analysis revealed that LDH level, ISS stage, extramedullary disease, and pre-ASCT were independent factors for predicting OS in patients with NDMM.^[29]^ In this study, age ≥ 65 years, ISS stage III, no ASCT, high β_2_-MG levels, high LDH levels, and high UA levels had prognostic effects, which is consistent with the results of the above studies. However, according to data from the GBD 2021 study,^[10]^ the age-standardized prevalence of MM is significantly higher in males than in females in China. Research^[34]^ has identified a positive correlation between increased male mortality and variables including obesity, per capita GDP, and the human development index.

There are also limitations in this study. First, this study is a single-center retrospective study. Second, the absence of baseline data (including imaging, blood biochemistry, and FISH results) should not be ignored. Our hospital began routine FISH testing in MM patients in 2016; the proportion of patients undergoing this test increased in 2018, and more than two-thirds of the missing values came from patients diagnosed before 2016, likely due to the high cost of the method. Therefore, there may be information bias in the analysis of prognostic factors. However, this change may further reflect the dynamic changes in NDMM management in southern Jiangxi Province. Third, because the survival status of some patients was determined by telephone follow-up, there may be measurement errors and misclassification of mortality. Fourth, ASCT was 1st carried out at our center in 2022, and few patients have undergone ASCT treatment; in addition, few patients receiving PIs + IMiDs as the main chemotherapy regimen have died. The follow-up time was limited, meaning that the curative effect could not be fully observed, and a longer follow-up time is needed.

## 5. Conclusions

In this study, we described the progressive improvements made by a provincial medical center in southern Gannan, China, over the past 10 years to refine its MM diagnosis and treatment model. With the popularization of new technologies and the widespread use of the latest standardized treatment protocols, the OS of MM patients in southern Gannan has improved significantly in the near and long term. However, there is still much room for improvement in the diagnosis and treatment of MM in resource-constrained areas.

## Author contributions

**Conceptualization:** Qi Xiao, Jing Li, Xiqing Rao, Sizhe Zhu.

**Data curation:** Qi Xiao.

**Formal analysis:** Qi Xiao, Pingfang Liu, Lunwang Liang, Sizhe Zhu.

**Funding acquisition:** Pingfang Liu, Zoufang Huang.

**Investigation:** Jing Li, Xiqing Rao.

**Methodology:** Pingfang Liu, Zoufang Huang.

**Project administration:** Qi Xiao, Zoufang Huang.

**Resources:** Lunwang Liang, Zoufang Huang.

**Software:** Pingfang Liu.

**Writing – original draft:** Qi Xiao, Pingfang Liu.

**Writing – review & editing:** Qi Xiao, Pingfang Liu, Zoufang Huang.
